# A novel large deletion of the *CYLD* gene causes *CYLD* cutaneous syndrome in a Chinese family

**DOI:** 10.1002/mgg3.1441

**Published:** 2020-08-11

**Authors:** Ruizheng Zhu, Jie Xu, Juan Shen, Wenru Li, Fei Tan, Changchang Li, Zhichen Wei, Yeqiang Liu, Yun Bai

**Affiliations:** ^1^ Shanghai Skin Disease Hospital Tongji University School of Medicine Shanghai China; ^2^ Wenzhou Hospital of Integrated Traditional Chinese and Western Medicine Wenzhou City China

**Keywords:** *CYLD* cutaneous syndrome, *CYLD* gene, large deletion, multiple familial trichoepithelioma, mutation

## Abstract

**Background:**

*CYLD* cutaneous syndrome (CCS; syn. Brooke‐Spiegler syndrome) is a rare autosomal dominant hereditary disease characterized by multiple adnexal skin tumors including cylindromas, spiradenomas, and trichoepitheliomas. More than 100 germline mutations of the cylindromatosis (*CYLD*) gene have been reported in CCS and most of them are frameshift mutations or small alterations.

**Methods:**

We identified a large, three‐generation Chinese family with CCS, which consisted of 18 living family members, including six affected individuals. To explore the molecular biology of this family, we carried out targeted next‐generation sequencing and Affymetrix CytoScan HD SNP array to analyze the mutation in the *CYLD* gene.

**Results:**

A novel large deletion mutation, NC_000016.9:g.(50826498_50827517)_(50963389‐50967346)del was found in the proband of this family. This deletion results in the loss of a nearly 140 kb fragment of the *CYLD* gene, spanning exons 17 ~ 20, which represent the coding regions of the ubiquitin‐specific protease domain. Further quantitative polymerase chain reaction proved that all patients and two proband‐related family members carried this large deletion.

**Conclusions:**

Our study expands the types of mutations in CCS and will undoubtedly provide valuable information for genetic counseling for families affected by the condition.

## Graphical abstract

A large, three‐generation Chinese family with CCS, which consisted of 18 living family members, including six affected individuals was identified. We carried out targeted next‐generation sequencing and Affymetrix CytoScan HD SNP array to analyze the mutation in the CYLD gene and a novel large deletion mutation was found.

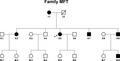

## 1 INTRODUCTION

*CYLD* (OMIM: 605018) cutaneous syndrome (CCS; syn. Brooke‐Spiegler syndrome) is a rare inherited skin tumor syndrome that encompasses the clinical phenotypes described in individuals with germline pathogenic *CYLD* variants. It was previously considered a group of diseases, including Brooke‐Spiegler syndrome (BSS; OMIM: 605041), familial cylindromatosis (FC; OMIM: 132700), and multiple familial trichoepithelioma (MFT; OMIM: 601606) on the basis of the predominant tumor type and location (Parren, Giehl, van Geel, & Frank, 2018). These disorders are now considered to constitute a clinical spectrum (Lee, Grossman, Schneiderman, & Celebi, 2005; Oranje et al., 2008; Parren et al., 2018). Individuals with the clinical phenotypes of BSS, FC, and MFT may simultaneously occur in some rare familial cases, suggesting that they may possess a common genetic origin (Bowen et al., 2005; Parren et al., 2018). Typically, MFT accounts for about 30% of all features of CCS (Nagy, Farkas, Kemeny, & Szell, 2015). It is characterized by the presence of multiple translucent, skin‐colored, hemispherical papules, and nodules around the perinasals, nasolabial folds, nose, forehead, and upper lips (Johnson & Bennett, 1993). Histopathologically, the condition is characterized by basaloid cells with peripheral palisades, which are arranged in nests or cribriform patterns surrounded by dense stroma and fibroblasts (Lian & Cockerell, 2005).

In 2004, Zhang et al. first identified the *CYLD* gene on chromosome 16q12‐13 as the pathogenic gene responsible for MFT (Zhang et al., 2004). *CYLD* is thought to be a tumor suppressor gene, which contains 20 exons and encodes a 120 kDa evolutionarily conserved protein (Brummelkamp, Nijman, Dirac, & Bernards, 2003). Up to date, more than 100 germline mutations of the *CYLD* gene have been identified in patients with CCS, including missense, insertion, deletion, or splice‐site mutations (Verhoeft, Ngan, & Lui, 2016).

In the current study, we recruited a Chinese CCS family of 18 members. Genetic analyses, including next‐generation sequencing (NGS) and quantitative real‐time polymerase chain reaction (qPCR) were performed, which led to the discovery of a novel large deletion mutation from exon 17 to exon 20 of the *CYLD* gene.

## 2 MATERIALS AND METHODS

### 2.1 Patients and ethical statement

**Figure 1:**
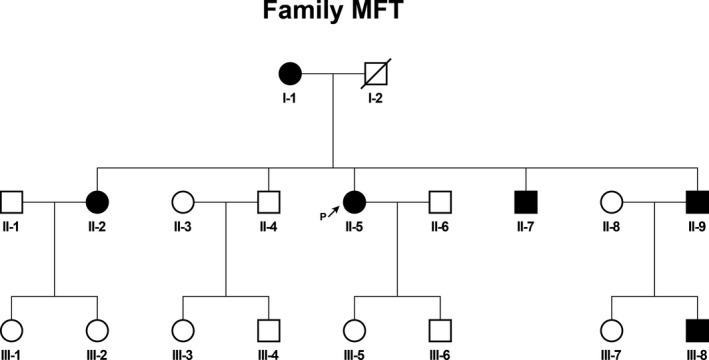
Pedigree of the CCS family. Family members with CCS are indicated with solid shading. Squares and circles denoted males and females, respectively. Individuals labeled with a solidus were deceased. Arrow indicates the proband

**Figure 2:**
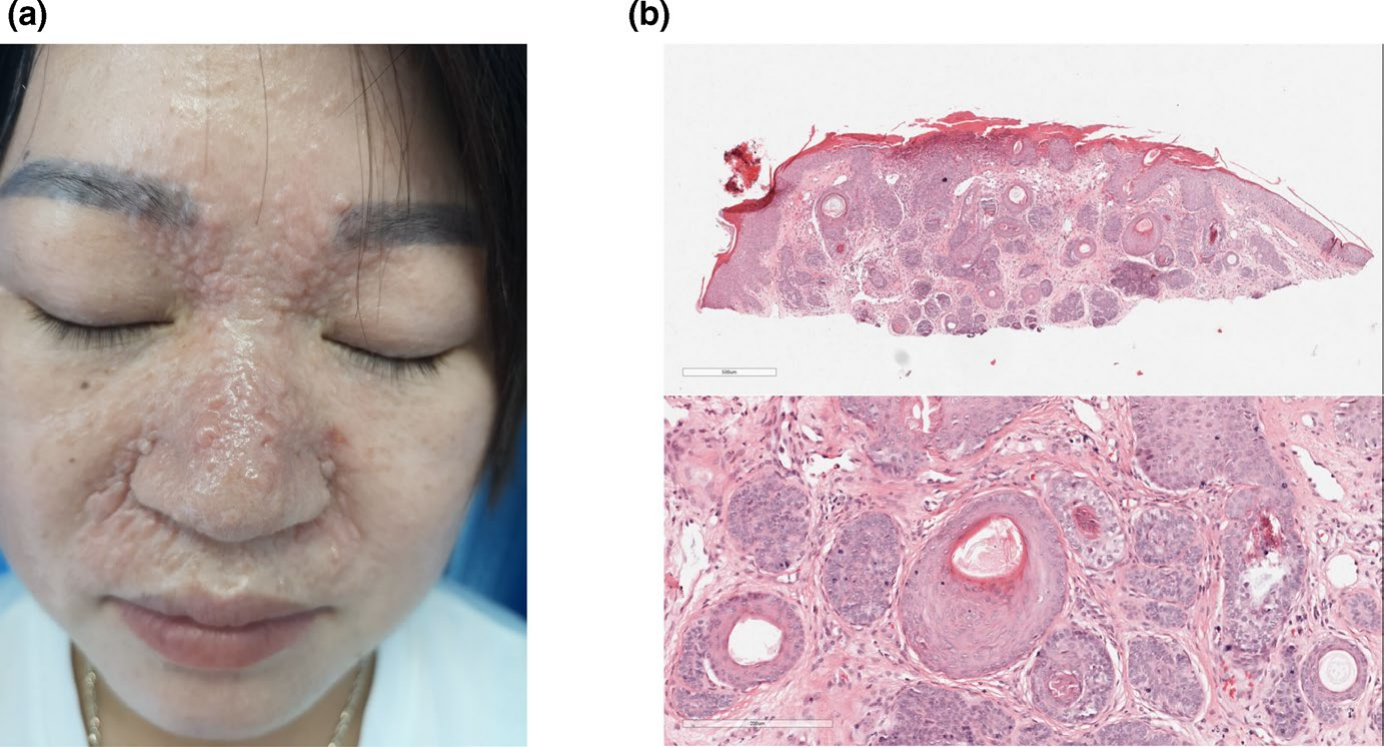
Clinical and histopathological features of the proband. (a) The picture showed the proband with multiple discrete and confluent skin‐colored papules and nodules located on the face, especially in nasolabial folds and inner aspects of eyebrows. (b) The figure showed the histopathological features of the proband. The neoplasm was composed of several fibroepithelial units, in which basaloid cells formed in a fibrous stroma with follicular germs and papillae

A three‐generation CCS family with a predominant MFT phenotype, consisting of 18 individuals (eight male and ten female), diagnosed and treated in the Wenzhou Hospital of Integrated Chinese Traditional and Western Medicine, was recruited in the study (Figure 1, Figure S1). The proband was a 40‐year‐old female, who presented a 25‐year history of multiple skin‐colored papules and nodules on the nose, nasolabial folds, bilateral cheeks, and forehead (Figure 2a). The papules and nodules gradually enlarged in size and increased in number with the patient's age. The clinical diagnosis of CCS was made by a dermatopathologist and was based on a combination of clinical characteristics and typical histopathological features (Figure 2b). All directly‐related members of this three‐generation Chinese family (six male and six female), who participated in this study provided written informed consent. The project which was in compliance with the Helsinki declaration was approved by the Ethical Committee of Wenzhou Hospital, China (Wenzhou, China).

### 2.2 DNA isolation and next‐generation sequencing

The genomic DNA was extracted from 200 µl of ethylenediaminetetraacetic acid (EDTA)‐treated peripheral blood using QIAamp DNA Blood Mini Kit (cat. no. 51304, Qiagen, Valencia, CA, USA), according to the manufacturer's instructions. The DNA concentration and purity were measured utilizing a NanoDrop 1000 spectrophotometer (Thermo Scientific, Waltham, USA). A total of 1 µg of DNA from the proband was employed to generate a genomic DNA library according to the protocols suggested by Illumina. Subsequently, a custom‐targeted capture kit from NimbleGen (Roche, Madison, USA) was designed to capture the 2 M region containing all exons of the 596 genes known to be associated with the common hereditary skin diseases, including CCS. Following the capture and PCR amplification, the library was sequenced by Illumina HiSeq X Ten analyzers (Illumina, San Diego, USA). The image analysis and base calling were conducted using the Illumina real‐time analysis (RTA) Pipeline software, version 1.9.

### 2.3 Bioinformatics analysis

After removing low quality reads, the remaining ones were aligned to the standard human genome reference (hg19) using the Burrows‐Wheeler analysis with default parameters. Subsequently, following the removal of duplicate reads, the alignment file was calculated for the reads corresponding to the targeted capture region. SNPs and INDELs were identified using GATK (http://www.broadinstitute.org/gatk/). CNV were detected as described in previous reports (Schiessl, Huettel, Kuehn, Reinhardt, & Snowdon, 2017).

### 2.4 Microarray analysis

To validate the CNV detected in the *CYLD* gene (NCBI Reference Sequence: NG_012061.1), the microarray analysis was performed using high‐density CytoScan microarray (Affymetrix, USA). The array contained 2.67 million markers, which allowed for the determination of the structural variants of more than 36,000 genes. A total of 1 µg of DNA from the proband was utilized to perform this microarray analysis according to the manufacturer's protocol suggested by Affymetrix. Following the procedures of sample preparation, hybridization, and scanning, the CEL file was analyzed by the Chromosome Analysis Suite 2.0 software (Affymetrix, USA). Notably, the software is specific for the analysis of microarray results from the CytoScan^TM^ HD Array.

### 2.5 Quantitative real‐time PCR for the validation of the large deletion of the *CYLD* gene

As the large deletion of the *CYLD* gene was detected by NGS and microarray in the proband, qPCR was performed to validate this copy number variation in the other members of the studied family. qPCR was carried out using SYBR Premix Ex Taq^TM^ (Takara Bio Inc., Tokyo, Japan) in a LightCycler 480 real‐time platform (Roche, Basel, Switzerland). Two exons in the deletion region were randomly selected (exon 18 and 20) to validate the obtained results. To evaluate the influence of the DNA concentration, two exons in the normal regions were also randomly selected (exons 4 and 9) as references. The primers were designed based on one of the transcripts from the *CYLD* gene (NM_015247). The primer sequences are listed in Table S1. For each PCR, a dissociation curve analysis was carried out to discriminate the specific products from the primer dimers. The CT values were the average of three technical and three biological replicates. The relative gene copy number was assessed by the 2^−ΔΔCt^ method.

## 3 RESULTS

### 3.1 Germline mutation analysis of peripheral blood by NGS

A custom NGS panel targeted sequencing was used to screen the pathological mutation in more than 596 genes related to common hereditary dermatosis to screen the pathological mutation of the proband, including the *CYLD* gene. This yielded nearly 4 million reads, with approximately 40% mapping to the targeted regions. The average sequencing depth of the target area was more than 250× with more than 99% coverage. The coverage and depth effectively reflected the genetic variation in the captured gene regions. Following SNPs calling and data filtering, no rare variants (frequency <0.01) in this patient appeared to be pathogenetic or in accord with the observed clinical phenotype (Table S2). However, considering strong clinical suspicion of CCS based on the immunohistochemical results, the NGS data were re‐evaluated, focusing on the *CYLD* gene. Checking the coverage obtained from the NGS data revealed a significant decrease in read counts on the last four exons, which suggested a large deletion, NC_000016.9:g. (50826498_50827517)_ (50963389‐50967346)del spanning exon 17 to 20 of the *CYLD* gene in this proband (Figure S2). The raw reads were deposited in the National Center for Biotechnology Information Sequence Read Archive with the accession number PRJNA645783.

### 3.2 Validation of the large deletion by CytoScan microarray

**Figure 3:**
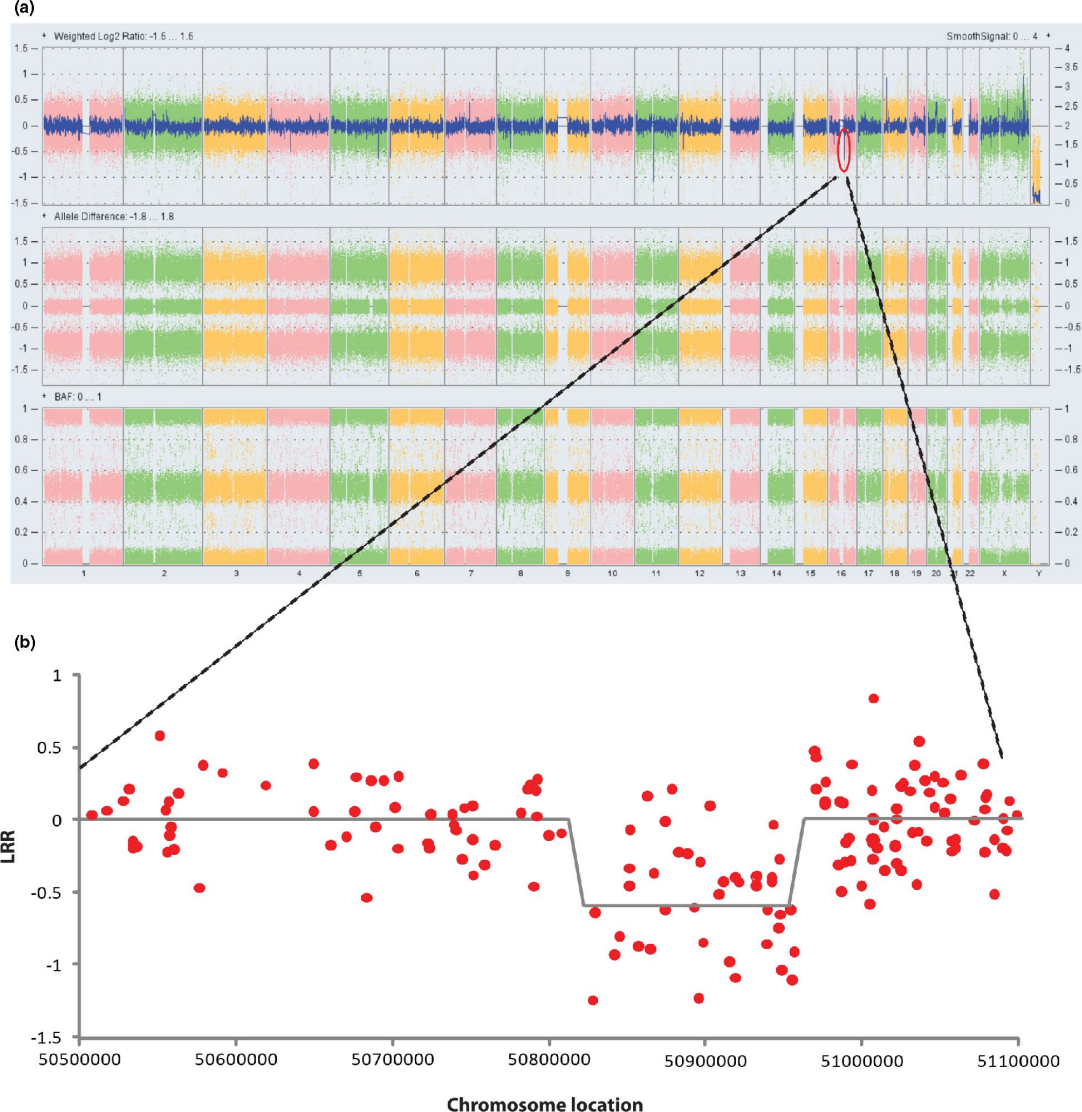
The log R ratios detected at the proband by microarray. (a) The log R ratios (LRR) and B allele frequencies (BAF) at the whole genome region. (b) The LRR at the *CYLD* deletion region. The *X* axis showed a part of Chromosome 16 (chr16: 50500000‐51100000). The *Y* axis showed the LRR of this part of the chromosome

To confirm the large deletion, a copy number analysis was carried out using high‐density CytoScan microarray (Affymetrix, USA). Fifteen CNV sites (six loss and nine gain) were identified in the patient. A significant copy number decrease was observed in chr16: 50,827,517‐50,967,346. This result further confirmed the findings of NGS (Figure 3, Table S3).

### 3.3 qPCR confirmation of NGS in other family members

**Figure 4:**
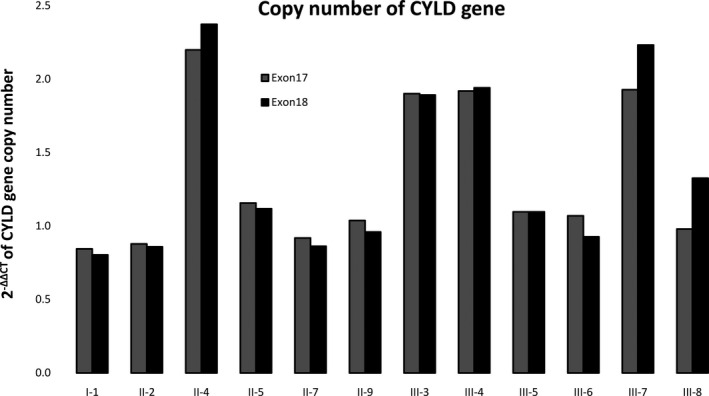
Validation of copy numbers of the CYLD gene by qPCR. The figure showed copy numbers of exons 18 and 20 quantified by QPCR. In order to adjust the influence of DNA concentration, we also randomly selected exon 9 in the normal regions as references. As the results, the proband (II‐5) and patients (I‐1, II‐2, II‐7, II‐9, III‐8), as well as two children of the proband (III‐5, III‐6), had only one copy at exons 18 and 20, while four unaffected family members (II‐4, III‐3, III‐4, III‐7) had nearly two copies

Since the large deletion was identified by NGS, qPCR was conducted to determine whether the variant existed in the other five affected family members. As shown in Figure 4, all affected family members (I‐1, II‐2, II‐5, II‐7, II‐9, III‐8) carried this large deletion. Furthermore, six unaffected family members (II‐4, III‐3, III‐4, III‐5, III‐6, III‐7) were also determined. It was found that most of them (II‐4, III‐3, III‐4, III‐7) did not carry the variant, except III‐5 and III‐6. These two children were related to the proband, suggesting that they may carry the large deletion mutation in the *CYLD* gene and may eventually have the disease in the future.

## 4 DISCUSSION

Various germline mutations in the *CYLD* gene have been identified as the causative pathogeny in CCS. In 2015, Nagy et al. investigated a total of 95 mutations in the *CYLD* gene from BSS, MFT, and FC. It was established that nearly 99% of the mutations were located within exons 9–20. The majority of the sequence changes were frameshift (48%), nonsense (27%), missense (12%), and splice‐site (11%) (Nagy et al., 2015). To date, a few studies have reported that patients with CCS exhibit large germline deletions in the *CYLD* gene. Out of four patients assessed in these studies, one with BSS exhibited an approximately 5.3 kB deletion at the 3′ end of the gene (Bignell et al., 2000). The second patient with BSS displayed an approximately 13.5 kB deletion from exon 6 ~ 12, while the third subject with MFT had a more extensive deletion, amounting to near‐complete omittance of the *CYLD* gene (Vanecek et al., 2014). The fourth patient with FC showed a 5.5 Mb deletion encompassing *CYLD* and 23 other genes (Davies et al., 2019). In the present study, we identified a novel large deletion mutation in a Chinese CCS family by NGS and microarray. It resulted in the omittance of four exons at the 3′ end of the *CYLD* gene. The results of our investigations suggested that large deletion mutations in the *CYLD* gene were also important types of hereditary variants in BSS or MFT.

A cohort study analyzed the associations between the subgroups of patients with a different phenotype and type of mutations. Nevertheless, no statistically significant correlation between the genotypes and phenotypes was established (Nagy et al., 2015). In the current study, we identified a large deletion of the last four exons in the *CYLD* gene in a CCS family with a predominant MFT phenotype. A similar deletion position has previously been reported in the last exon, which was derived from a patient with BSS (Vanecek et al., 2014). These conflicting results further confirmed that the locations or subtypes of the germline variants in the *CYLD* gene are not the only reasons for the complex disease phenotypes. Some alterations in other genes or noncoding regions with regulatory functions may interact with the mutant CYLD protein, and thus, also determine the disease phenotypes. Recently, an integrated genomic, methylation, and transcriptomic analysis demonstrated the existence of recurrent mutations in the epigenetic modifiers *DNMT3A* and *BCOR* in the patient with CCS (Davies et al., 2019). Furthermore, long noncoding RNA, such as CRAL and GMDS‐AS1, is suspected to regulate the expression of miR‐505 or miR‐96‐5p, and the latter has been shown to inhibit the expression of the *CYLD* gene (Wang et al., 2020; Zhao et al., 2020).

In the present study, a novel germline deletion of the last four exons was identified. This copy number variation of the *CYLD* gene may affect the expression of the ubiquitin‐specific protease (USP) domain of the CYLD protein, which is likely to be responsible for the abnormal clinical manifestations in the CCS patients. A study by Massoumi et al. used genetically‐engineered mouse models and demonstrated that the loss of a single copy of the *CYLD* gene was sufficient to confer a multiple tumor phenotype upon chemical compound‐induced mice. The incidence and growth rates of tumors in *CYLD*+/‐ mice significantly increased and were in‐between those of *CYLD*+/+ and *CYLD*‐/‐, indicating a potential dose effect of the *CYLD* gene on the proliferation of tumor cells (Masoumi, Shaw‐Hallgren, & Massoumi, 2011). Moreover, a considerable overexpression of MYB was observed in the CYLD‐defective cylindroma cells, while the knockdown of MYB expression caused a significant reduction in the proliferation of these cells (Rajan et al., 2016). These phenomena suggested that copy number variations in the *CYLD* gene may also be key players and oncogenic drivers in inherited skin tumors.

In conclusion, in the current study, we evaluated a novel large deletion mutation of the *CYLD* gene in a large Chinese pedigree, which was determined to play a critical role in the tumorigenesis and progression of CCS. The results of our study expand the spectrum of the *CYLD* mutations and provide valuable information for the development of new treatments or prevention of this disfiguring tumor.

## CONFLICT OF INTEREST

The authors declare that the research was conducted in the absence of any commercial or financial relationships that could be construed as a potential conflict of interest.

## AUTHOR CONTRIBUTIONS

Ruizheng Zhu and Jie Xu coordinated the project. Jie Xu and Juan Shen participated in the blood sample preparation and DNA extraction. Wenru Li, Fei Tan, and Changchang Li directed and performed NGS and SNP microarray. Yun Bai, Ruizheng Zhu, and Zhichen Wei carried out the quantitative RT‐PCR. Yun Bai and Ruizheng Zhu performed the data analyses and wrote the manuscript. Yeqiang Liu helped perform the analysis with constructive discussions.

## Supporting information

Fig S1

Fig S2

Table S1

Table S2

Table S3
