# Supplementary figures and images for: A novel large deletion of the CYLD gene causes CYLD cutaneous syndrome in a Chinese family
Source: Mol Genet Genomic Med. 2020 Aug 11;8(10):e1441. doi: 10.1002/mgg3.1441 (PMC7549610; doi:10.1002/mgg3.1441)

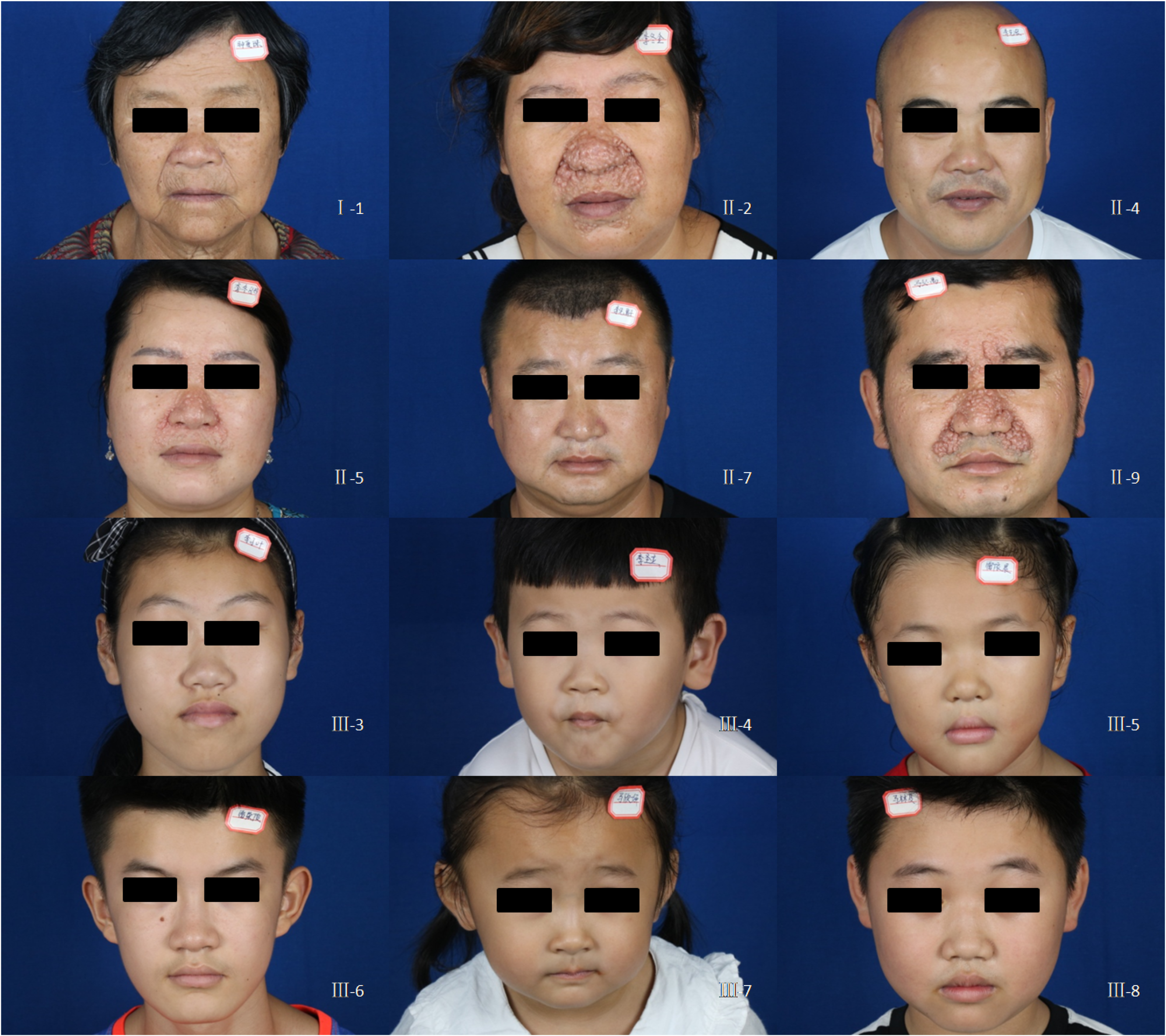

Supplement: Supplementary file 1 — Fig S1 [file MGG3-8-e1441-s001.png]

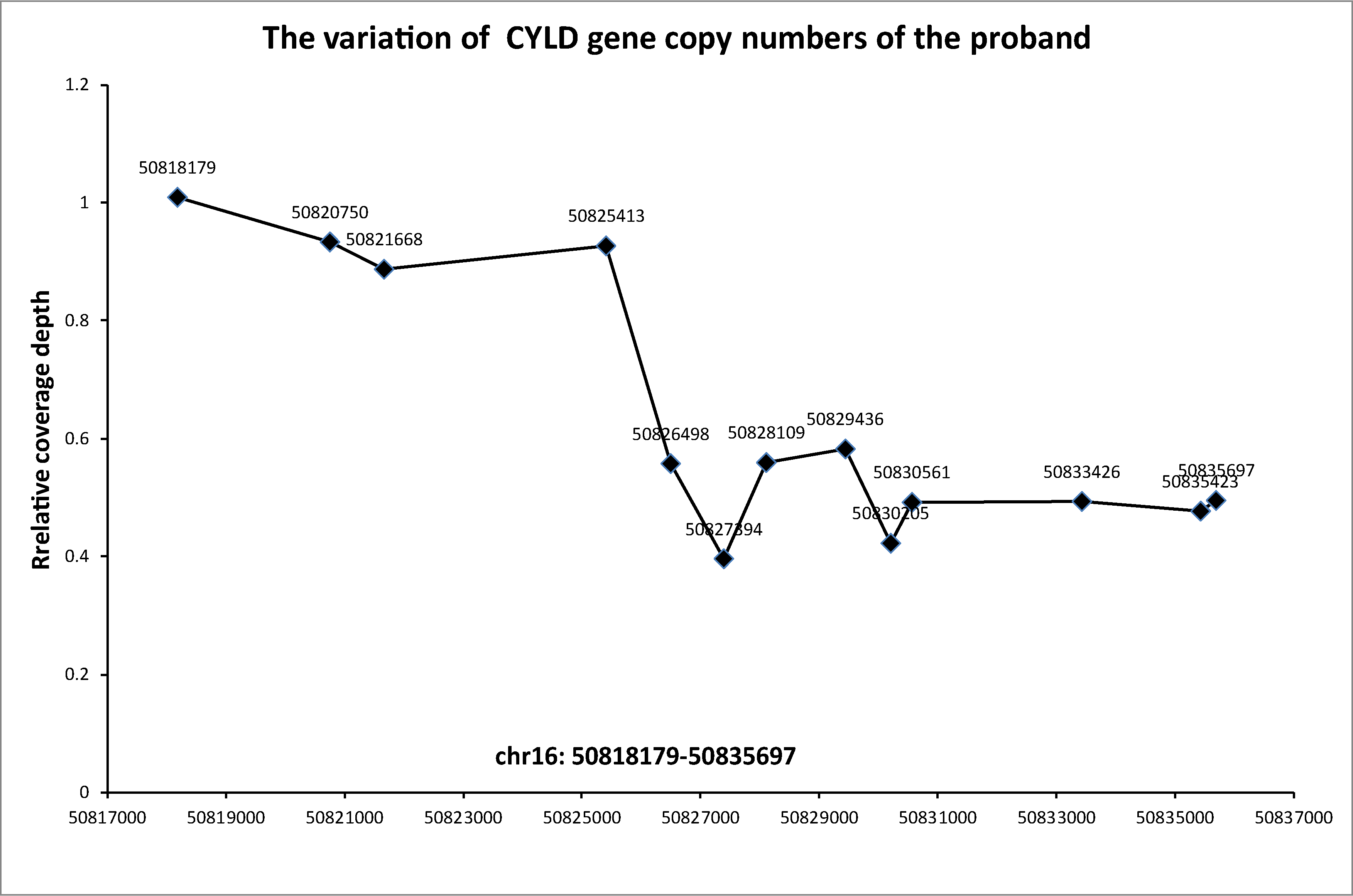

Supplement: Supplementary file 2 — Fig S2 [file MGG3-8-e1441-s002.png]
